# Motivation Theories and Constructs in Experimental Studies of Online Instruction: Systematic Review and Directed Content Analysis

**DOI:** 10.2196/64179

**Published:** 2025-04-11

**Authors:** Adam Gavarkovs, Erin Miller, Jaimie Coleman, Tharsiga Gunasegaran, Rashmi A Kusurkar, Kulamakan Kulasegaram, Melanie Anderson, Ryan Brydges

**Affiliations:** 1 Division of Continuing Professional Development Faculty of Medicine University of British Columbia Vancouver, BC Canada; 2 School of Physical Therapy Faculty of Health Sciences Western University London, ON Canada; 3 School of Physical Therapy University of Toronto Toronto, ON Canada; 4 University of Toronto Toronto, ON Canada; 5 Amsterdam UMC location Vrije Universiteit Amsterdam Amsterdam The Netherlands; 6 Department of Family and Community Medicine Temerty Faculty of Medicine University of Toronto Toronto, ON Canada; 7 University Health Network Toronto, ON Canada; 8 Department of Medicine Temerty Faculty of Medicine University of Toronto Toronto, ON Canada

**Keywords:** motivation, internet, systematic review, experimental studies, online instruction, educator, learner, researcher, health professional, education, tool-kit, autonomy

## Abstract

**Background:**

The motivational design of online instruction is critical in influencing learners’ motivation. Given the multifaceted and situated nature of motivation, educators need access to a range of evidence-based motivational design strategies that target different motivational constructs (eg, interest or confidence).

**Objective:**

This systematic review and directed content analysis aimed to catalog the motivational constructs targeted in experimental studies of online motivational design strategies in health professions education. Identifying which motivational constructs have been most frequently targeted by design strategies—and which remain under-studied—can offer valuable insights into potential areas for future research.

**Methods:**

Medline, Embase, Emcare, PsycINFO, ERIC, and Web of Science were searched from 1990 to August 2022. Studies were included if they compared online instructional design strategies intending to support a motivational construct (eg, interest) or motivation in general among learners in licensed health professions. Two team members independently screened and coded the studies, focusing on the motivational theories that researchers used and the motivational constructs targeted by their design strategies. Motivational constructs were coded into the following categories: intrinsic value beliefs, extrinsic value beliefs, competence and control beliefs, social connectedness, autonomy, and goals.

**Results:**

From 10,584 records, 46 studies were included. Half of the studies (n=23) tested strategies aimed at making instruction more interesting, enjoyable, and fun (n=23), while fewer studies tested strategies aimed at influencing extrinsic value beliefs (n=9), competence and control beliefs (n=6), social connectedness (n=4), or autonomy (n=2). A focus on intrinsic value beliefs was particularly evident in studies not informed by a theory of motivation.

**Conclusions:**

Most research in health professions education has focused on motivating learners by making online instruction more interesting, enjoyable, and fun. We recommend that future research expand this focus to include other motivational constructs, such as relevance, confidence, and autonomy. Investigating design strategies that influence these constructs would help generate a broader toolkit of strategies for educators to support learners’ motivation in online settings.

**Trial Registration:**

PROSPERO CRD42022359521; https://www.crd.york.ac.uk/PROSPERO/view/CRD42022359521

## Introduction

The internet has become a preferred modality for health professions education (HPE) in the postpandemic landscape [1]. A recent global survey found that 60% of health professionals preferred blended learning, while 32% preferred fully online learning [2]. Online instruction can ameliorate barriers due to geography, scheduling, and cost that make in-person learning infeasible for many health professionals and trainees [3]. However, one challenge of online learning is keeping learners motivated. Motivation—the energetic force that instigates and sustains behavior [4]—is key to success when learning online [5,6]. A lack of face-to-face interaction and the metacognitive demands associated with learning online can lead to feelings of isolation, frustration, and diminished motivation [7,8]. To address these challenges and keep learners motivated, educators must build motivational support into online instruction through a process known as motivational design [9].

Motivational design is defined by Keller [9] as “the process of arranging resources and procedures to bring about changes in people’s motivation.” This process involves selecting, adapting, and applying motivational design strategies, which are resources and procedures that facilitate the motivational processes underpinning learning. For example, Colonnello et al [10] enhanced medical students’ motivation by supplementing surgical videos with emotionally salient patient information. Other studies have demonstrated that other motivational design strategies, such as using narration in online modules, can impact learner motivation [11,12].

Motivational design strategies work by influencing various motivational constructs—cognitive factors that shape learners’ moment-to-moment motivation [4]. Broad categories of motivational constructs include goals (“What am I aiming to do?”), competence beliefs (“Can I do it?”), value beliefs (“Do I want to do it? Why?”), and attributional beliefs (“Why did it happen this way?”) [13]. For example, an educator might use a strategy to make learning seem more relevant, increase learners’ interest, or boost their confidence that they can learn the material.

Theories of motivation emphasize that learners’ motivation is influenced by several motivational constructs, any one of which may be the cause of poor motivation during online learning [4]. For example, medical students completing an online module on a basic science topic may be confident in their ability to learn but struggle to see the value in the material beyond their next examination. Conversely, students completing a virtual examination with a standardized patient may see the value in what they are learning but not feel confident in their ability to succeed. In the first case, an educator could use a strategy that targets learners’ value beliefs (eg, a prompt to reflect on the clinical relevance of the material [14]), while in the second, an educator could use a strategy that targets’ learners competence beliefs (eg, providing a demonstration that learners can observe beforehand [9]). Given the multifaceted and situated nature of motivation, educators need access to a range of evidence-based motivational design strategies that target different motivational constructs, such as strategies for enhancing confidence or perceived value [15].

Researchers can support educators by providing evidence on the effectiveness of different motivational design strategies [16]. However, we do not have a good understanding of which motivational constructs are most frequently targeted in research on online motivational design. For example, are researchers disproportionately focused on testing ways to make online instruction more interesting or enjoyable? An expanding literature on serious games and gamification in HPE suggests this may be the case, as games are often framed as a strategy to enhance interest [17-24]. While enhancing interest is important, if researchers focus too narrowly on this construct at the expense of others (eg, confidence), then educators may not receive the full range of design strategies needed to support learner motivation [4]. To inform future research, it is important to identify which motivational constructs have been most emphasized and which remain under-studied.

To address this gap, our review aims to catalog the motivational constructs targeted in studies of online motivational design strategies. This is a novel objective, as no previous reviews have organized the instructional design literature based on the motivational constructs that strategies aim to influence. By identifying which constructs have received the most attention, we aim to guide future literature syntheses on the most effective design strategies for supporting these constructs. Additionally, by identifying under-studied constructs, we aim to guide areas for future primary research. Ultimately, our review is intended as a resource for researchers interested in conducting future studies on motivational design for online instruction. Stimulating ongoing research in this area will ensure that educators have access to evidence-based guidance to design more motivating online instruction.

We hypothesize that there are two reasons why certain motivational constructs may be underrepresented in research on online motivational design strategies: (1) studies are not informed by a theory of motivation or model of motivational design, or (2) studies are informed by such theories but choose not to focus on specific constructs. To disentangle these explanations, we posed two research questions: (1) Which theories or models of motivation, if any, inform experimental comparison studies of motivational design strategies for online instruction? (2) Within experimental comparison studies of motivational design strategies for online instruction, which motivational constructs, if any, have been targeted?

## Methods

We conducted a systematic review and directed content analysis focused on experimental comparison studies in HPE [25]. Experimental comparison studies, which compare 1 version of online instruction to another, are uniquely positioned to generate empirical evidence for the causal effects of motivational design strategies [25-28]. Motivational design is, at its core, a process of making predictions about the causal effects of motivational design strategies (“If I use this strategy, will it cause my learners to be more motivated?”). Since experimental comparison studies are best suited for making causal claims, we consider them a necessary source of evidence for educators and serve as the focus for our review. Bajpai et al [29] adopted a similar position in their recent review of learning theories in randomized trials of digital instruction in HPE.

Given our focus on experimental comparison studies, we identified a systematic review as the most appropriate review methodology [30]. We registered (PROSPERO CRD42022359521) and published a review protocol [31], and report our findings in accordance with the PRISMA (Preferred Reporting Items for Systematic Reviews and Meta-Analyses) 2020 updated guidelines [32], with a few exceptions. We omit items 12 (effect measures), 14 (reporting bias assessment), 15 (certainty assessment), 19 (results of individual studies), 21 (risk of bias due to missing results), and 22 (certainty of evidence), as we did not intend to appraise nor synthesize the outcomes of included studies. Further details on our methods can be found in our published protocol [31]. To increase the clarity and brevity of reporting, this paper omits data related to a few research questions listed in our published protocol. Additional data regarding these questions is available upon request.

### Eligibility Criteria

#### Study Characteristics

We included individual and cluster randomized controlled trials and quasi-experimental studies published in English from 1990 to August 2, 2022 (for databases) and September 15, 2022 (for registries). Our date range aligns with prior reviews of digital education in HPE [33]. We included protocols for planned or ongoing studies but excluded conference abstracts and unpublished studies. Studies were not excluded based on quality or risk of bias as we did not aim to synthesize the results of studies. However, we appraised the risk of bias to provide readers with additional context regarding the quality of studies.

#### Participants

We included studies focusing on learners in the health professions regardless of training status (see protocol for list of health professions), either exclusively or when mixed with other learners (eg, psychology students).

#### Interventions

We included studies comparing online instructional designs (or that could have been delivered online, such as CD-ROM instruction), which targeted a motivational construct (eg, interest) or motivation more generally. By “targeting” motivation, we mean that researchers stated that their instructional design aimed to enhance learner motivation to engage with instruction. Several studies demonstrated a cursory treatment of motivation, for example, by discussing the impact of design strategies on constructs (eg, interest) without grounding the construct in a theoretical framework. We decided to include these studies because they contribute to our understanding of the foci among researchers interested in this area of HPE. Studies comparing online instruction against paper-based or face-to-face instruction were excluded.

#### Outcomes

We included studies that assessed any learner outcome.

### Search Strategy and Selection Process

#### Database Searching

Strategies were developed for Ovid Medline, Embase, Emcare PsycINFO, EBSCO ERIC, and Web of Science Core Collection (Social Sciences Citation Index; Arts & Humanities Citation Index; Book Citation Index-Social Sciences & Humanities; Conference Proceedings Citation Index-Science; Emerging Sources Citation Index; Science Citation Index; Book Citation Index-Social Sciences & Humanities; and Conference Proceedings Citation Index-Social Science & Humanities) by a health sciences librarian (MA) in collaboration with the review team (Multimedia Appendix 1). Appropriate subject headings and keywords for motivation, online instruction, and HPE focused on the licensed professions were used for each database. The results were limited to those published from 1990 to the date of the searches. The searches were run on August 2, 2022, and the 14,736 results were uploaded to Covidence for screening.

#### Registry Searching

For the Open Science Framework Registries, we developed 12 searches, comprised of different combinations of the highest yielding terms in our database searches (Multimedia Appendix 2). The searches yielded between 7277 and 16,018 hits for each combination of terms. AG manually screened the first 10 pages of results (10 results per page) for each search (1200 studies screened in total) and uploaded 19 potentially relevant studies to Covidence.

#### Hand and Reference Searching

AG manually screened several published literature reviews on online instruction in HPE [18-23,34-39] and the references of included studies and uploaded 161 potentially relevant studies to Covidence.

#### Screening

After removing duplicates, we screened 10,584 records. Two team members independently screened abstracts and, as necessary, the paper’s full text. Before independent screening, all 6 team members who participated in the screening process practiced screening the same 30 abstracts, and then discussed and refined the inclusion criteria. AG also developed a decision tool to support full-text screening. As screening progressed, AG periodically reviewed conflicts for any systematic issues and further refined the inclusion and exclusion criteria. Two senior team members (EM or RB) not involved in the initial decision resolved all conflicts. We included 61 studies in the data extraction phase. During the extraction phase we excluded an additional 15 studies. In 12 cases, the papers were excluded because they did not discuss the potential motivational effects of a strategy in the introduction or did not state an objective to assess the effects of a strategy on motivation. Therefore, we concluded that these were not motivational design strategies [40-51]. This yielded 46 studies included in our review.

### Data Collection and Synthesis Methods

#### Overview

The data items we extracted can be found in Multimedia Appendix 3. We conducted a directed content analysis during the extraction process [52], coding each study deductively regarding the motivational theories used and the motivational constructs targeted. We piloted and refined the extraction process in Covidence with a few included studies. AG trained team members to extract and code data. Two team members independently extracted data from each study. Conflicts were resolved through discussion, with an experienced team member (ie, currently in, or having completed, a PhD program) not involved in the initial decision leading to resolution.

#### Theories of Motivation (Aligned With Research Question 1)

We developed an a priori list of 6 prominent theories of motivation and 1 model of motivational design to deductively guide our coding. We defined theories as “prominent” based on meeting one of the following criteria: (1) they were included in a 2020 special issue of *Contemporary Educational Psychology* titled “Prominent Motivation Theories: The Past, Present, and Future” [53-57], or (2) they have been the subject of an AMEE Guide in *Medical Teacher* [58,59]. We also added Keller’s ARCS model of motivational design, which we assumed would be cited in HPE studies [24]. Brief descriptions of these theories can be found in Table 1. Beyond this initial list, we considered any theory aiming to explain the energetic basis and direction of learners’ engagement to be a theory of motivation [60]. We also coded whether these theories informed 4 key aspects of the research process: the research questions, the design of the experimental conditions, the selection of methods and measures, and the interpretation of results [61].

**Table 1:** Overview of and reported use of established theories of motivation and models of motivational design.

Theory or model	Description	Frequency used, n (%)	References
SDT^a^ [55]	Ryan and Deci’s SDT differentiates between types of motivation depending on learners’ reasons for engaging in learning, such as feeling pressured to satisfy external demands (external regulation), feeling pressured to quell feelings of guilt or shame (introjected regulation), identifying with the value of an activity (identified regulation), or finding the activity inherently interesting (intrinsic motivation). SDT also emphasizes the influence of the social environment on learners’ motivation, as mediated by the satisfaction of feelings of autonomy (ie, being in control of one’s actions), competence (ie, feeling efficacious in one’s actions), and relatedness (ie, feeling connected to others).	8 (17)	[10,11,62-67]
ARCS^b^ model [9]	Keller’s ARCS model states that, for learners to become and remain motivated to learn, their attention must be captured via feelings of curiosity, they must perceive instruction to be relevant to their current needs and long-term goals, they must feel confident that they can succeed, and they must feel satisfied with the intrinsic and extrinsic consequences of engaging with instruction.	6 (13)	[5,68-72]
SCT^c^ [56]	Bandura’s SCT emphasizes the primary role of learners’ self-efficacy beliefs (ie, that they can execute courses of action needed to attain particular outcomes) and outcome expectancies (ie, that courses of action will lead to particular outcomes) in motivating their learning goal pursuit.	3 (7)	[64,73,74]
CVT^d^ [75]	Pekrun’s CVT posits that the achievement emotions that learners experience (as well as their self-regulation and learning) are most proximally a function of the subjective control and value beliefs they ascribe to actions and outcomes for an activity. Subjective control beliefs are based on action-control expectations (ie, expectations that actions can be performed) and action-outcome expectations (ie, expectations that particular actions will lead to certain outcomes). Subjective value beliefs are based on the perceived intrinsic and extrinsic value of engaging in the activity and attaining resultant outcomes.	2 (4)	[10,63]
EVT^e^ [53]	Eccles and Wigfield’s EVT (now called situated expectancy-value theory) posits that learners’ motivation is most proximally a function of their expectations of success and the subjective value they ascribe to an activity. Subjective value is composed of interest value (ie, the interest or enjoyment an activity brings), utility value (ie, an activity’s usefulness for attaining other valued goals), attainment value (ie, an activity’s importance in confirming a salient aspect of one’s identity), and cost (ie, the drawbacks of completing an activity).	1 (2)	[76]
Other theories or models	Theory of narrative engagement [77,78]; 4-phase model of interest development [11]; engagement modes model [73]; information and communication acceptance model [79]; social interdependence theory [80]; Guthrie and Wigfield engagement model [81]	N/A^f^	See description
None mentioned	N/A	24 (52)	[82-105]

^a^SDT: self-determination theory.

^b^ARCS: attention, relevance, confidence, and satisfaction.

^c^SCT: social cognitive theory.

^d^CVT: control-value theory.

^e^EVT: expectancy-value theory

^f^N/A: not applicable.

#### Motivational Constructs (Aligned With Research Question 2)

We used our list of theories and previous research [13] to create a priori categories of motivational constructs to deductively guide our coding. During the coding process, our categorization scheme changed slightly from that documented in our protocol [31], as we determined that a more parsimonious categorization scheme involved aggregating more constructs into fewer categories (Multimedia Appendix 4). Our list included the following categories of motivational constructs: intrinsic value beliefs (eg, interest), extrinsic value beliefs (eg, instrumentality), competence and control beliefs (eg, self-efficacy), social connectedness (eg, relatedness), autonomy, and goals. Intrinsic value refers to the value derived from the experience of completing an activity (eg, interest or enjoyment), whereas extrinsic value refers to the value derived from attaining outcomes external to an activity (eg, progress toward future goals) [53,55].

#### Study Risk of Bias Assessment

We rated each study’s risk of bias across 9 dimensions contained within the Cochrane Collaboration’s Effective Practice and Organization of Care risk of bias tool: random sequence generation, allocation concealment, similar baseline outcome measurements, similar baseline characteristics, incomplete outcome data, blinded outcome measurement, protection against contamination, selective outcome reporting, and other risks of bias [30]. This tool has been used in similar systematic reviews of online instruction in HPE [19,36]. Team members reported particular difficulty in identifying “other risks of bias,” and we observed that raters frequently documented different sources of bias (or no bias) within this broad category. Accordingly, we decided to exclude this dimension.

## Results

### Characteristics of Included Studies

**Figure 1:**
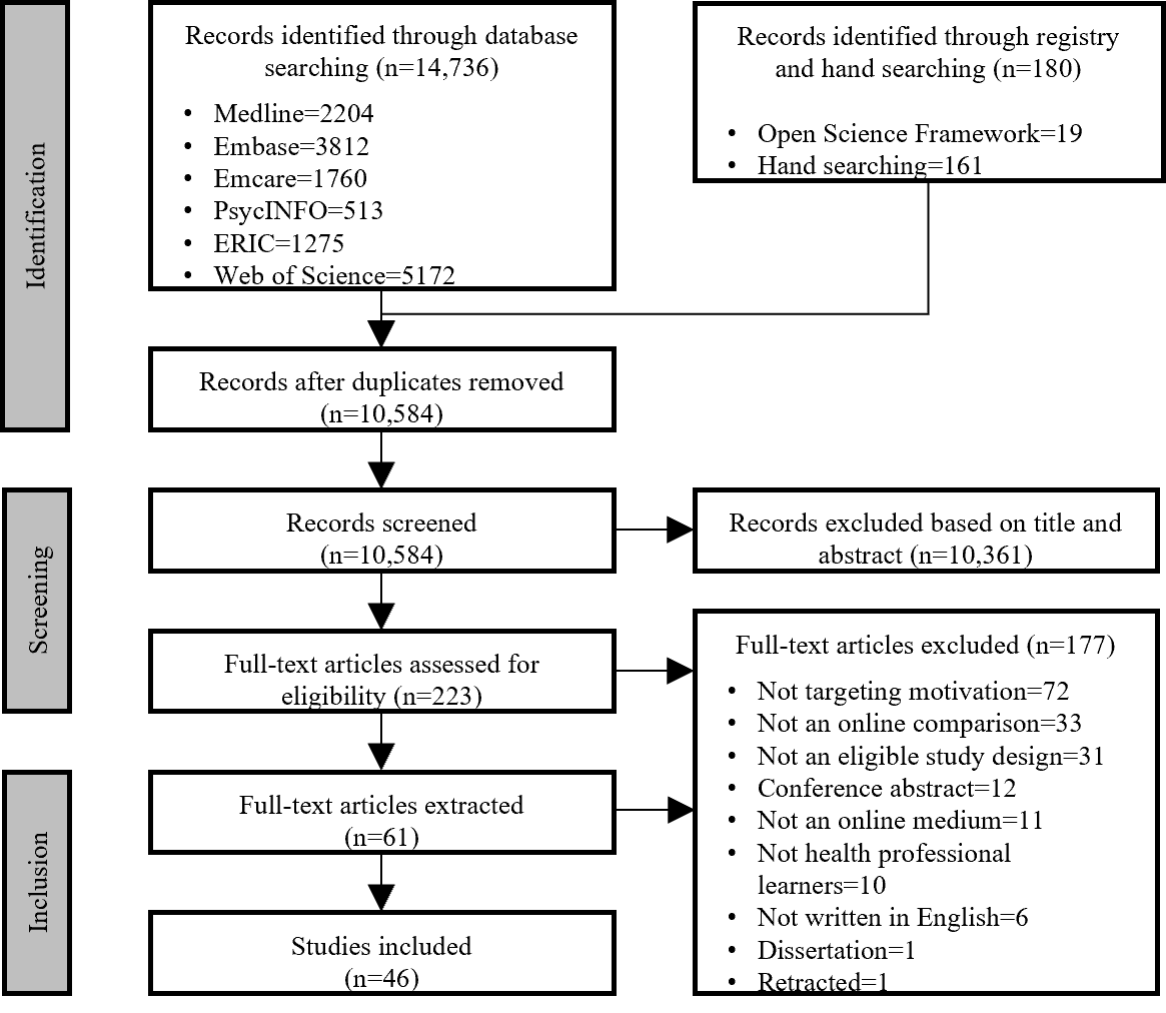
PRISMA flowchart. PRISMA: Preferred Reporting Items for Systematic Reviews and Meta-Analyses.

The characteristics of the included studies are presented in Multimedia Appendix 5. Most studies were conducted with trainees (n=40), primarily medical students (n=17) and nursing students (n=11). Study designs were predominantly randomized parallel-group trials (n=27), followed by quasi-experimental trials (n=12), randomized cross-over trials (n=4), and cluster randomized trials (n=3). The risks of bias for each study are presented in Multimedia Appendix 6. Although 74% (34/46) of the included studies were identified as randomized trials, only 30% (14/46) were rated as low risk of bias for random sequence generation, and 33% (15/46) were rated as low risk of bias for allocation concealment. For other dimensions of bias, low risk was observed in 35% (16/46) of studies for baseline outcome measurements, 37% (17/46) for baseline characteristics, 50% (23/46) for blinded outcome measurements, 50% (23/46) for contamination, 57% (26/46) for missing outcome data, and 80% (37/46) for selective outcome reporting. The PRISMA flowchart for our review is presented in Figure 1, and the PRISMA checklist can be found in Multimedia Appendix 7.

### Which Theories or Models of Motivation Inform Existing Experimental Studies of Motivational Design Strategies?

Table 1 presents the number of studies that were informed by a theory of motivation or model of motivational design. SDT and the ARCS model were the most commonly used theories, while 24 studies did not cite any theory. Five studies cited more than 1 theory of motivation. Among the 22 studies that used at least 1 theory, we judged the theory as informing the research questions in 20 (91%) studies, informing the experimental conditions in 15 (68%) studies, informing methods and measures in 17 (77%) studies, and informing the interpretation of results in 17 (77%) studies. Nine studies used theory to inform all 4 aspects of their research process [5,10,11,62,63,68,69,77,80].

### Which Motivational Constructs Have Studies Targeted With Their Motivational Design Strategies?

Studies investigated motivational design strategies that targeted intrinsic value beliefs in 23 of the 46 (50%) studies, extrinsic value beliefs in 9 (20%) studies, competence and control beliefs in 6 (13%) studies, social connectedness in 4 (9%) studies, and autonomy in 2 (4%) studies. Ten (22%) studies targeted more than 1 construct; of these, 5 (11%) were informed by the ARCS model. Sixteen (35%) studies did not report targeting any specific motivational construct, instead aiming to enhance motivation in general.

While intrinsic value beliefs were the most commonly targeted construct, researchers drawing on a prominent theory or model (as listed in Table 1) tended to be more pluralistic in their foci. Specifically, studies that used a motivation theory or model targeted intrinsic value beliefs (n=11) at a similar level to extrinsic value beliefs (n=9) and, to a lesser extent, competence and control beliefs (n=6). By contrast, studies that did not use a theory or model focused solely on intrinsic value beliefs (n=10) compared to extrinsic value beliefs (n=0) and competence and control beliefs (n=0).

## Discussion

### Key Findings and Implications for Future Research

In this systematic review, we analyzed experimental comparison studies of online motivational design strategies in HPE. We aimed to identify which motivational constructs have been most frequently targeted in these studies and which remain understudied, offering insights into potential areas for future research.

A significant finding was that nearly one-third of the studies in our review did not specify which motivational constructs their design strategy was targeting, instead broadly aiming to enhance motivation. We argue that such research is of limited value to educators. Motivational design expertise relies on educators understanding how strategies work, specifically what constructs they influence and under what conditions they are most effective [106,107]. Studies that do not clarify which constructs a design strategy influences, either conceptually or empirically, cannot provide educators with the information needed to build expertise [16]. Therefore, we recommend that researchers explicitly define the motivational constructs their strategies aim to influence and test their impact on those constructs. This recommendation can be supported through the greater use of motivational theories, which were cited in fewer than half of the studies in our review. This lack of theory use is consistent with other reviews, such as those by Maheu-Cadotte et al [19] and Bajpai et al [29], who found similarly low levels of theory use in their reviews of serious games and digital education in HPE. Motivational theory should be used to inform the research questions, the design strategy, the outcome measures, and the interpretation of results. Excellent examples of theory use are present in our sample [5,11,80].

Among the studies that did specify targeted constructs, most focused on intrinsic value beliefs (eg, interest or enjoyment), compared to extrinsic value beliefs, competence and control beliefs, social connectedness, and autonomy. Accordingly, research in this area is disproportionately focused on ways to make online instruction more interesting and enjoyable. Given the volume of studies on design strategies targeting intrinsic value beliefs, we recommend that future research synthesize existing findings to identify the most effective strategies for enhancing interest and enjoyment and outline areas for future research.

A disproportionate focus on enhancing intrinsic value beliefs aligns with an increased uptake of SDT in HPE, as documented in our studies and other reviews [24,108]. SDT emphasizes the role of intrinsic motivation—which is grounded in feelings of interest and enjoyment—in effective learning [55]. However, we found that studies using SDT were often pluralistic in the constructs they targeted, suggesting a more nuanced approach than studies without a theoretical basis. A theoretical perspective, whether based on SDT or another theory, may help researchers avoid equating motivation solely with enjoyment and interest, thus neglecting other facets of motivation, such as confidence and relatedness, despite evidence suggesting that these constructs may be particularly at risk when learning online [7,8]. Supporting this perspective, we found that studies informed by the ARCS model—which explicitly states the importance of supporting learners’ attention, relevance, confidence, and satisfaction—were most likely to report targeting multiple motivational constructs. We recommend that studies test design strategies targeting a broader range of motivational constructs to expand the set of design strategies that educators can choose from (eg, confidence-enhancing strategies or relatedness-enhancing strategies). For example, though serious games are often framed as ways to enhance interest and enjoyment, they may also be configured to support feelings of practical relevance or boost confidence [24]. Researchers could build on the serious games literature by investigating ways to design serious games to support feelings of extrinsic value, confidence, social connectedness, and autonomy.

We encourage researchers to study ways of motivating learners in established online modalities (eg, asynchronous modules or webinars) and by using emerging technologies such as virtual reality and artificial intelligence. For example, artificial intelligence chatbots have the potential to provide personalized coaching and feedback during learning [109,110]. Providing such support and scaffolding instruction in a learner’s zone of proximal development may foster a sense of autonomy and confidence. As research on the motivational design of emerging online modalities is still in its infancy, future studies could investigate how to design emerging technology-enabled instruction to optimize learner motivation.

The risk of bias was a concern across many of the included studies. To ensure that future research can make more defendable claims regarding the effects of design strategies, researchers should clearly specify procedures for random sequence generation and allocation concealment, which are often missing from published papers. They should also capture relevant variables at baseline, blind assessors to condition, and attempt to limit attrition and contamination [27].

### Limitations

Several limitations are worth noting. We did not include any synonyms for the word “motivation” (eg, “engagement” or “satisfaction”) or motivational constructs (eg, “value,” “relevance,” or “confidence”) in our search terms because we believed these terms would greatly increase the number of nonrelevant studies in our search results. We assumed that studies using synonyms for “motivation” or referencing motivational constructs would also use the word “motivation” and thus would be retrieved in our searches. Consequently, we may have missed some otherwise eligible studies that exclusively referenced concepts that are related to, or treated as synonymous to, motivation (eg, engagement) or motivational constructs (eg, confidence). We also chose to exclude studies written in a language other than English, which may have resulted in missed studies.

We decided to focus our review on experimental studies because they provide a critical source of evidence regarding the effectiveness of design strategies. We acknowledge that many different kinds of studies can generate evidence to support educators’ motivational design efforts when producing online learning [31,111]. For example, qualitative studies can help us understand how learners make meaning of instructional designs in context [112], and single-group studies can investigate the factors influencing engagement with motivational design strategies [113]. It may be that studies leveraging nonexperimental designs demonstrate a different distribution of foci regarding motivational constructs. We recommend that a breadth of methodologies, including but not limited to experimental comparison studies, be used to investigate novel motivational design strategies in the future.

Finally, our review focused on online instruction in HPE, and it is unclear whether the trends we observed apply to other types of HPE, such as in-person simulation. While the trend toward enhancing interest and enjoyment may also be present in other HPE contexts—such as through the gamification of in-person instruction [114-116]—we cannot make definitive claims about the generalizability of our results to other types of HPE. Conducting similar reviews in other areas of HPE may be a focus of future research.

### Conclusions

A key challenge for educators when teaching online involves keeping learners motivated. To address this challenge, educators need access to motivational design strategies that target a range of motivational constructs. The existing research provides an important starting point, but there is much work to be done. Researchers can use our findings to guide future primary and secondary research that generates a more robust evidence base for educators wishing to motivate their learners.

## Appendix

Multimedia Appendix 1 Database search strategies.

Multimedia Appendix 2 Registry search strategy.

Multimedia Appendix 3 Data items.

Multimedia Appendix 4 Categories of motivational constructs.

Multimedia Appendix 5 Characteristics of included studies.

Multimedia Appendix 6 Risk of bias ratings for included studies.

Multimedia Appendix 7 PRISMA 2020 checklist. PRISMA: Preferred Reporting Items for Systematic Reviews and Meta-Analyses.

## Abbreviations

HPE: health professions education
PRISMA: Preferred Reporting Items for Systematic Reviews and Meta-Analyses
