# Supplementary material for: Motivation Theories and Constructs in Experimental Studies of Online Instruction: Systematic Review and Directed Content Analysis
Source: JMIR Med Educ. 2025 Apr 11;11:e64179. doi: 10.2196/64179 (PMC12032500; doi:10.2196/64179)
Supplement: Multimedia Appendix 1 [file mededu_v11i1e64179_app1.docx]

## Searches run August 2, 2022 in:

- Ovid Medline(R) ALL 1946 to August 01, 2022
- Ovid Embase 1974 to 2022 August 01
- Ovid Emcare Nursing 1995 to Present
- Ovid APA PsycInfo 1806 to July Week 4 2022
- EBSCOhost Research Databases - ERIC
- Web of Science (SSCI, AHCI, BHCI, ISTP, ESCI, SCI, BSCI, ISSHP)

**Ovid Medline(R) ALL 1946 to August 01, 2022**

| **Line** | **Search terms** | **Results** |
| --- | --- | --- |
| 1 | Computer-Assisted Instruction/ | 12434 |
| 2 | exp video-audio media/ | 37927 |
| 3 | (elearn* or e-learn*).mp. | 4361 |
| 4 | internet-based.mp. | 11010 |
| 5 | web-based.mp. | 39312 |
| 6 | virtual.mp. | 84078 |
| 7 | online.mp. | 187103 |
| 8 | computer-based.mp. | 15088 |
| 9 | net-based.mp. | 519 |
| 10 | webinar*.mp. | 1364 |
| 11 | video?.mp. | 177140 |
| 12 | (recorded or recording?).mp. | 815223 |
| 13 | learning management system?.mp. | 441 |
| 14 | digital??.mp. | 191347 |
| 15 | (Blackboard Learn or CERTPOINT or D2L or Desire2Learn or Brightspace or eCollege or Edmodo or EduNxt or Engrade or GlobalScholar or (Glow adj20 Scott*) or HotChalk or Kahoot or Kannu or SAP or Skillsoft or Spongelab or SuccessFactors or "SumTotal Systems" or Taleo or (Teams adj20 microsoft) or Uzity or aTutor or Chamilo or Claroline or Canvas or eFront or ILIAS or LAMS or "LON CAPA" or Moodle or "Open edX" or OLAT or OpenOLAT or Sakai or SWAD or WeBWorK or "e khool" or CallidusCloud or "Cornerstone OnDemand" or DoceboLMS or eFront or EthosCE or "Google Classroom?" or Grovo or "Growth Engineering" or "Halogen Software" or "Inquisiq R3" or "Learning Suite" or itslearning or Kannu or OpenLearning or Udutu or "ANGEL Learning" or Click2Learn or CourseInfo or "Learn com" or Elluminate or PeopleSoft or "Plateau Systems" or Softscape or SuccessFactors or WebCT or Instructure or Oracle or Docent or Zoom or coursera or futurelearn or iversity or "khan academy" or udemy or swayam or "mit ocw" or openclassroom?).mp. [list of LMS and online learning platforms] | 22165 |
| 16 | edutech.mp. | 1 |
| 17 | (synchronous or asynchronous).mp. | 50082 |
| 18 | webcast*.mp. | 1782 |
| 19 | screencast*.mp. | 61 |
| 20 | (screenshar* or screen-shar*).mp. | 62 |
| 21 | (mlearn* or m-learn*).mp. | 113 |
| 22 | web-conferenc*.mp. | 160 |
| 23 | computer simulation*.mp. | 221019 |
| 24 | cd-rom.mp. | 1757 |
| 25 | (vhs? or dvd?).mp. | 3767 |
| 26 | or/1-25 | 1666556 |
| 27 | Motivation/ | 76704 |
| 28 | motivat*.ti,kf. | 29472 |
| 29 | (motivat* adj4 (student* or learner* or staff* or educat* or trainee* or instructee* or user? or pupil* or mentee* or tutee* or antendee* or participant*)).ab. | 9572 |
| 30 | or/27-29 | 93424 |
| 31 | 26 and 30 | 9300 |
| 32 | (teach* or educat* or academic).jw. | 177671 |
| 33 | education, predental/ or education, premedical/ or exp education, professional/ or exp inservice training/ or exp schools, health occupations/ or professional development/ | 368570 |
| 34 | exp Students, Health Occupations/ | 82766 |
| 35 | patient simulation/ | 5437 |
| 36 | or/32-35 | 495639 |
| 37 | exp Teaching/ | 91779 |
| 38 | exp curriculum/ | 95077 |
| 39 | clinical competence/ | 102824 |
| 40 | professional competence/ | 25023 |
| 41 | pedagog*.mp. | 11548 |
| 42 | ed.fs. | 296309 |
| 43 | (student * or learner* or learned or learns or teach* or educat* or instruct* or class or classes or workshop? or module? or train??? or curricul* or upskill* or retrain??? or webinar* or elearn* or e-learn* or in-service or inservice or professional develop* or continuing professional or CPD or CME or life-long-learn* or lifelong* learn* or certificate program? or certification?).ti,kw,hw. | 1082130 |
| 44 | educat*.mp. | 1162474 |
| 45 | competen*.ti,kf,hw. | 166392 |
| 46 | or/37-45 | 1605318 |
| 47 | exp health personnel/ | 588451 |
| 48 | exp health occupations/ | 1818741 |
| 49 | (health* adj4 (personnel or occupation* or profession*)).mp. | 396105 |
| 50 | (medic* or physician* or doctor* or p?ediatric* or allergist* or anesthesiologist* or dermatologist* or radiologist* or general practitioner* or geneticist* or neurologist* or obstetrician* or gyn?ecologist* or ophthalmologist* or pathologist* or oncologist* or urologist* or surgeon* or nurs* or therapist* or dent* or pharmac* or allied health* or physiotherapist* or psychiatrist* or psychologist* or audiolog* or chirop* or podiatr* or chiropractor* or dietition* or dietetic* or hearing aid* or homeopath* or RMT or medic* laborator* or medical radiat* or radiographer* or midwif* or midwiv* or naturopath* or osteopath* or operating department* or optician* or optometr* or orthodont* or orthopt* or paramed* or prosthetic* or pedorthist* or orthotist* or respirator* or speech language or social work* or traditional chinese medic* or acupunctur*).mp. | 9826092 |
| 51 | (hospital* adj4 (personnel or staff)).mp. | 99698 |
| 52 | (clinic* adj4 (personnel or staff)).mp. | 12989 |
| 53 | or/47-52 | 10546386 |
| 54 | 46 and 53 | 972284 |
| 55 | 36 or 54 | 1095294 |
| 56 | 31 and 55 | 2241 |
| 57 | limit 56 to yr="1990 -Current" | 2204 |

**Ovid Embase 1974 to 2022 August 01**

| **Line** | **Search terms** | **Results** |
| --- | --- | --- |
| 1 | educational technology/ | 3309 |
| 2 | videoconferencing/ | 7287 |
| 3 | web conferencing/ | 305 |
| 4 | webcast/ | 422 |
| 5 | webinar/ | 1052 |
| 6 | computer simulation/ | 132425 |
| 7 | exp videorecording/ | 106138 |
| 8 | exp CD-ROM/ | 42 |
| 9 | (elearn* or e-learn*).mp. | 9358 |
| 10 | internet-based.mp. | 13580 |
| 11 | web-based.mp. | 55055 |
| 12 | virtual.mp. | 112223 |
| 13 | online.mp. | 266467 |
| 14 | computer-based.mp. | 20635 |
| 15 | net-based.mp. | 627 |
| 16 | webinar*.mp. | 2815 |
| 17 | video?.mp. | 193669 |
| 18 | (recorded or recording?).mp. | 1099458 |
| 19 | learning management system?.mp. | 618 |
| 20 | digital??.mp. | 274802 |
| 21 | (Blackboard Learn or CERTPOINT or D2L or Desire2Learn or Brightspace or eCollege or Edmodo or EduNxt or Engrade or GlobalScholar or (Glow adj20 Scott*) or HotChalk or Kahoot or Kannu or SAP or Skillsoft or Spongelab or SuccessFactors or "SumTotal Systems" or Taleo or (Teams adj20 microsoft) or Uzity or aTutor or Chamilo or Claroline or Canvas or eFront or ILIAS or LAMS or "LON CAPA" or Moodle or "Open edX" or OLAT or OpenOLAT or Sakai or SWAD or WeBWorK or "e khool" or CallidusCloud or "Cornerstone OnDemand" or DoceboLMS or eFront or EthosCE or "Google Classroom?" or Grovo or "Growth Engineering" or "Halogen Software" or "Inquisiq R3" or "Learning Suite" or itslearning or Kannu or OpenLearning or Udutu or "ANGEL Learning" or Click2Learn or CourseInfo or "Learn com" or Elluminate or PeopleSoft or "Plateau Systems" or Softscape or SuccessFactors or WebCT or Instructure or Oracle or Docent or Zoom or coursera or futurelearn or iversity or "khan academy" or udemy or swayam or "mit ocw" or openclassroom?).mp. [list of LMS and online learning platforms] | 30314 |
| 22 | edutech.mp. | 0 |
| 23 | (synchronous or asynchronous).mp. | 62606 |
| 24 | webcast*.mp. | 748 |
| 25 | screencast*.mp. | 84 |
| 26 | (screenshar* or screen-shar*).mp. | 114 |
| 27 | (mlearn* or m-learn*).mp. | 128 |
| 28 | web-conferenc*.mp. | 529 |
| 29 | computer simulation*.mp. | 141089 |
| 30 | cd-rom.mp. | 1413 |
| 31 | (vhs? or dvd?).mp. | 5626 |
| 32 | or/1-31 | 2126115 |
| 33 | motivation/ or extrinsic motivation/ or intrinsic motivation/ or motivational intensity/ | 117078 |
| 34 | motivat*.ti,kf. | 33055 |
| 35 | (motivat* adj4 (student* or learner* or staff* or educat* or trainee* or instructee* or user? or pupil* or mentee* or tutee* or attendee* or participant*)).ab. | 11815 |
| 36 | 33 or 34 or 35 | 132725 |
| 37 | 32 and 36 | 15037 |
| 38 | exp health student/ | 129896 |
| 39 | exp medical education/ | 350375 |
| 40 | exp paramedical education/ | 96074 |
| 41 | medical school/ or pharmacy school/ | 67308 |
| 42 | exp interdisciplinary education/ or interprofessional education/ | 2950 |
| 43 | social work education/ | 94 |
| 44 | 38 or 39 or 40 or 41 or 42 or 43 | 500066 |
| 45 | exp teaching/ | 101687 |
| 46 | education/ | 456565 |
| 47 | continuing education/ | 32437 |
| 48 | curriculum/ or curriculum development/ | 103282 |
| 49 | educational model/ | 8769 |
| 50 | in service training/ | 15912 |
| 51 | clinical competence/ or nursing competence/ or professional competence/ | 100215 |
| 52 | pedagog*.mp. | 13477 |
| 53 | (student * or learner* or learned or learns or teach* or educat* or instruct* or class or classes or workshop? or module? or train??? or curricul* or upskill* or retrain??? or webinar* or elearn* or e-learn* or in-service or inservice or professional develop* or continuing professional or CPD or CME or life-long-learn* or lifelong* learn* or certificate program? or certification?).ti,kw,hw. | 1818755 |
| 54 | educat*.mp. | 1501714 |
| 55 | competen*.ti,kf,hw. | 165312 |
| 56 | 45 or 46 or 47 or 48 or 49 or 50 or 51 or 52 or 53 or 54 or 55 | 2195200 |
| 57 | medical profession/ or nursing as a profession/ or nursing career/ or paramedical profession/ | 26621 |
| 58 | exp health care personnel/ | 1812359 |
| 59 | (health* adj4 (personnel or occupation* or profession*)).mp. | 508412 |
| 60 | (medic* or physician* or doctor* or p?ediatric* or allergist* or anesthesiologist* or dermatologist* or radiologist* or general practitioner* or geneticist* or neurologist* or obstetrician* or gyn?ecologist* or ophthalmologist* or pathologist* or oncologist* or urologist* or surgeon* or nurs* or therapist* or dent* or pharmac* or allied health* or physiotherapist* or psychiatrist* or psychologist* or audiolog* or chirop* or podiatr* or chiropractor* or dietition* or dietetic* or hearing aid* or homeopath* or RMT or medic* laborator* or medical radiat* or radiographer* or midwif* or midwiv* or naturopath* or osteopath* or operating department* or optician* or optometr* or orthodont* or orthopt* or paramed* or prosthetic* or pedorthist* or orthotist* or respirator* or speech language or social work* or traditional chinese medic* or acupunctur*).mp. | 11908159 |
| 61 | (hospital* adj4 (personnel or staff)).mp. | 43021 |
| 62 | (clinic* adj4 (personnel or staff)).mp. | 20660 |
| 63 | 57 or 58 or 59 or 60 or 61 or 62 | 12177125 |
| 64 | 56 and 63 | 1340065 |
| 65 | 44 or 64 | 1397993 |
| 66 | 37 and 65 | 3828 |
| 67 | limit 66 to yr="1990 -Current" | 3812 |

**Ovid Emcare Nursing 1995 to Present**

| **Line** | **Search terms** | **Results** |
| --- | --- | --- |
| 1 | educational technology/ | 1904 |
| 2 | videoconferencing/ | 3004 |
| 3 | web conferencing/ | 47 |
| 4 | webcast/ | 84 |
| 5 | webinar/ | 174 |
| 6 | computer simulation/ | 15167 |
| 7 | exp videorecording/ | 35368 |
| 8 | exp CD-ROM/ | 25 |
| 9 | (elearn* or e-learn*).mp. | 4449 |
| 10 | internet-based.mp. | 6741 |
| 11 | web-based.mp. | 23120 |
| 12 | virtual.mp. | 37416 |
| 13 | online.mp. | 111463 |
| 14 | computer-based.mp. | 8591 |
| 15 | net-based.mp. | 197 |
| 16 | webinar*.mp. | 834 |
| 17 | video?.mp. | 56552 |
| 18 | (recorded or recording?).mp. | 238205 |
| 19 | learning management system?.mp. | 329 |
| 20 | digital??.mp. | 78724 |
| 21 | (Blackboard Learn or CERTPOINT or D2L or Desire2Learn or Brightspace or eCollege or Edmodo or EduNxt or Engrade or GlobalScholar or (Glow adj20 Scott*) or HotChalk or Kahoot or Kannu or SAP or Skillsoft or Spongelab or SuccessFactors or "SumTotal Systems" or Taleo or (Teams adj20 microsoft) or Uzity or aTutor or Chamilo or Claroline or Canvas or eFront or ILIAS or LAMS or "LON CAPA" or Moodle or "Open edX" or OLAT or OpenOLAT or Sakai or SWAD or WeBWorK or "e khool" or CallidusCloud or "Cornerstone OnDemand" or DoceboLMS or eFront or EthosCE or "Google Classroom?" or Grovo or "Growth Engineering" or "Halogen Software" or "Inquisiq R3" or "Learning Suite" or itslearning or Kannu or OpenLearning or Udutu or "ANGEL Learning" or Click2Learn or CourseInfo or "Learn com" or Elluminate or PeopleSoft or "Plateau Systems" or Softscape or SuccessFactors or WebCT or Instructure or Oracle or Docent or Zoom or coursera or futurelearn or iversity or "khan academy" or udemy or swayam or "mit ocw" or openclassroom?).mp. [list of LMS and online learning platforms] | 5198 |
| 22 | edutech.mp. | 0 |
| 23 | (synchronous or asynchronous).mp. | 9722 |
| 24 | webcast*.mp. | 147 |
| 25 | screencast*.mp. | 45 |
| 26 | (screenshar* or screen-shar*).mp. | 37 |
| 27 | (mlearn* or m-learn*).mp. | 108 |
| 28 | web-conferenc*.mp. | 141 |
| 29 | computer simulation*.mp. | 15960 |
| 30 | cd-rom.mp. | 680 |
| 31 | (vhs? or dvd?).mp. | 1384 |
| 32 | or/1-31 | 543008 |
| 33 | motivation/ or extrinsic motivation/ or intrinsic motivation/ or motivational intensity/ | 54314 |
| 34 | motivat*.ti,kf. | 19492 |
| 35 | (motivat* adj4 (student* or learner* or staff* or educat* or trainee* or instructee* or user? or pupil* or mentee* or tutee* or attendee* or participant*)).ab. | 6896 |
| 36 | 33 or 34 or 35 | 62391 |
| 37 | 32 and 36 | 7840 |
| 38 | exp health student/ | 59981 |
| 39 | exp medical education/ | 124321 |
| 40 | exp paramedical education/ | 38284 |
| 41 | medical school/ or pharmacy school/ | 22877 |
| 42 | exp interdisciplinary education/ or interprofessional education/ | 1662 |
| 43 | social work education/ | 1714 |
| 44 | 38 or 39 or 40 or 41 or 42 or 43 | 190511 |
| 45 | exp teaching/ | 52100 |
| 46 | education/ | 180926 |
| 47 | continuing education/ | 17908 |
| 48 | curriculum/ or curriculum development/ | 42477 |
| 49 | educational model/ | 1908 |
| 50 | in service training/ | 1821 |
| 51 | clinical competence/ or nursing competence/ or professional competence/ | 21706 |
| 52 | pedagog*.mp. | 9399 |
| 53 | (student * or learner* or learned or learns or teach* or educat* or instruct* or class or classes or workshop? or module? or train??? or curricul* or upskill* or retrain??? or webinar* or elearn* or e-learn* or in-service or inservice or professional develop* or continuing professional or CPD or CME or life-long-learn* or lifelong* learn* or certificate program? or certification?).ti,kw,hw. | 708720 |
| 54 | educat*.mp. | 596204 |
| 55 | competen*.ti,kf,hw. | 69043 |
| 56 | 45 or 46 or 47 or 48 or 49 or 50 or 51 or 52 or 53 or 54 or 55 | 845683 |
| 57 | medical profession/ or nursing as a profession/ or nursing career/ or paramedical profession/ | 10886 |
| 58 | exp health care personnel/ | 823051 |
| 59 | (health* adj4 (personnel or occupation* or profession*)).mp. | 219065 |
| 60 | (medic* or physician* or doctor* or p?ediatric* or allergist* or anesthesiologist* or dermatologist* or radiologist* or general practitioner* or geneticist* or neurologist* or obstetrician* or gyn?ecologist* or ophthalmologist* or pathologist* or oncologist* or urologist* or surgeon* or nurs* or therapist* or dent* or pharmac* or allied health* or physiotherapist* or psychiatrist* or psychologist* or audiolog* or chirop* or podiatr* or chiropractor* or dietition* or dietetic* or hearing aid* or homeopath* or RMT or medic* laborator* or medical radiat* or radiographer* or midwif* or midwiv* or naturopath* or osteopath* or operating department* or optician* or optometr* or orthodont* or orthopt* or paramed* or prosthetic* or pedorthist* or orthotist* or respirator* or speech language or social work* or traditional chinese medic* or acupunctur*).mp. | 2727994 |
| 61 | (hospital* adj4 (personnel or staff)).mp. | 14886 |
| 62 | (clinic* adj4 (personnel or staff)).mp. | 8131 |
| 63 | 57 or 58 or 59 or 60 or 61 or 62 | 2834957 |
| 64 | 56 and 63 | 513698 |
| 65 | 44 or 64 | 536893 |
| 66 | 37 and 65 | 1760 |
| 67 | limit 66 to yr="1990 -Current" | 1760 |

**Ovid APA PsycInfo 1806 to July Week 4 2022**

| **Line** | **Search terms** | **Results** |
| --- | --- | --- |
| 1 | exp computer assisted instruction/ | 22958 |
| 2 | virtual classrooms/ | 1019 |
| 3 | asynchronous learning/ | 67 |
| 4 | blended learning/ | 685 |
| 5 | exp audiovisual instruction/ | 2261 |
| 6 | learning management systems/ | 568 |
| 7 | computer simulation/ | 4758 |
| 8 | (elearn* or e-learn*).mp. | 4027 |
| 9 | internet-based.mp. | 6091 |
| 10 | web-based.mp. | 15563 |
| 11 | virtual.mp. | 29942 |
| 12 | online.mp. | 117151 |
| 13 | computer-based.mp. | 9522 |
| 14 | net-based.mp. | 70 |
| 15 | webinar*.mp. | 318 |
| 16 | video?.mp. | 55191 |
| 17 | (recorded or recording?).mp. | 156890 |
| 18 | learning management system?.mp. | 1151 |
| 19 | digital??.mp. | 39892 |
| 20 | (Blackboard Learn or CERTPOINT or D2L or Desire2Learn or Brightspace or eCollege or Edmodo or EduNxt or Engrade or GlobalScholar or (Glow adj20 Scott*) or HotChalk or Kahoot or Kannu or SAP or Skillsoft or Spongelab or SuccessFactors or "SumTotal Systems" or Taleo or (Teams adj20 microsoft) or Uzity or aTutor or Chamilo or Claroline or Canvas or eFront or ILIAS or LAMS or "LON CAPA" or Moodle or "Open edX" or OLAT or OpenOLAT or Sakai or SWAD or WeBWorK or "e khool" or CallidusCloud or "Cornerstone OnDemand" or DoceboLMS or eFront or EthosCE or "Google Classroom?" or Grovo or "Growth Engineering" or "Halogen Software" or "Inquisiq R3" or "Learning Suite" or itslearning or Kannu or OpenLearning or Udutu or "ANGEL Learning" or Click2Learn or CourseInfo or "Learn com" or Elluminate or PeopleSoft or "Plateau Systems" or Softscape or SuccessFactors or WebCT or Instructure or Oracle or Docent or Zoom or coursera or futurelearn or iversity or "khan academy" or udemy or swayam or "mit ocw" or openclassroom?).mp. [list of LMS and online learning platforms] | 3561 |
| 21 | edutech.mp. | 4 |
| 22 | (synchronous or asynchronous).mp. | 8461 |
| 23 | webcast*.mp. | 120 |
| 24 | screencast*.mp. | 82 |
| 25 | (screenshar* or screen-shar*).mp. | 27 |
| 26 | (mlearn* or m-learn*).mp. | 286 |
| 27 | web-conferenc*.mp. | 135 |
| 28 | computer simulation*.mp. | 15236 |
| 29 | cd-rom.mp. | 746 |
| 30 | (vhs? or dvd?).mp. | 1473 |
| 31 | or/1-30 | 405115 |
| 32 | motivation/ or exp achievement motivation/ or extrinsic motivation/ or intrinsic motivation/ or motivation measures/ | 74166 |
| 33 | motivat*.ti,id. | 71731 |
| 34 | (motivat* adj4 (student* or learner* or staff* or educat* or trainee* or instructee* or user? or pupil* or mentee* or tutee* or attendee* or participant*)).ab. | 16994 |
| 35 | 32 or 33 or 34 | 101485 |
| 36 | 31 and 35 | 10379 |
| 37 | dental students/ or medical students/ | 14610 |
| 38 | nursing students/ | 6056 |
| 39 | exp medical education/ | 25896 |
| 40 | nursing education/ | 6963 |
| 41 | dental education/ or exp graduate psychology education/ or rehabilitation education/ | 8393 |
| 42 | counselor education/ or paraprofessional education/ | 7166 |
| 43 | social work education/ | 5443 |
| 44 | or/37-43 | 62700 |
| 45 | exp teaching/ | 132567 |
| 46 | learning/ | 74015 |
| 47 | organizational learning/ | 4181 |
| 48 | problem based learning/ | 1507 |
| 49 | exp skill learning/ | 6055 |
| 50 | learning theory/ | 4830 |
| 51 | curriculum/ or curriculum development/ | 35149 |
| 52 | exp competence/ | 29536 |
| 53 | pedagog*.mp. | 38958 |
| 54 | (student * or learner* or learned or learns or teach* or educat* or instruct* or class or classes or workshop? or module? or train??? or curricul* or upskill* or retrain??? or webinar* or elearn* or e-learn* or in-service or inservice or professional develop* or continuing professional or CPD or CME or life-long-learn* or lifelong* learn* or certificate program? or certification?).ti,id,hw. | 707167 |
| 55 | educat*.mp. | 728557 |
| 56 | competen*.ti,id,hw. | 45686 |
| 57 | or/45-56 | 1041017 |
| 58 | exp social workers/ | 13977 |
| 59 | exp allied health personnel/ | 6354 |
| 60 | exp medical personnel/ | 90409 |
| 61 | exp mental health personnel/ | 55680 |
| 62 | health personnel/ | 19516 |
| 63 | clinicians/ | 12402 |
| 64 | (health* adj4 (personnel or occupation* or profession*)).mp. | 130786 |
| 65 | (medic* or physician* or doctor* or p?ediatric* or allergist* or anesthesiologist* or dermatologist* or radiologist* or general practitioner* or geneticist* or neurologist* or obstetrician* or gyn?ecologist* or ophthalmologist* or pathologist* or oncologist* or urologist* or surgeon* or nurs* or therapist* or dent* or pharmac* or allied health* or physiotherapist* or psychiatrist* or psychologist* or audiolog* or chirop* or podiatr* or chiropractor* or dietition* or dietetic* or hearing aid* or homeopath* or RMT or medic* laborator* or medical radiat* or radiographer* or midwif* or midwiv* or naturopath* or osteopath* or operating department* or optician* or optometr* or orthodont* or orthopt* or paramed* or prosthetic* or pedorthist* or orthotist* or respirator* or speech language or social work* or traditional chinese medic* or acupunctur*).mp. | 945119 |
| 66 | (hospital* adj4 (personnel or staff)).mp. | 11276 |
| 67 | (clinic* adj4 (personnel or staff)).mp. | 4151 |
| 68 | or/58-67 | 1013877 |
| 69 | 57 and 68 | 230295 |
| 70 | 44 or 69 | 243858 |
| 71 | 36 and 70 | 521 |
| 72 | limit 71 to yr="1990 -Current" | 513 |

| **EBSCOhost Research Databases - ERIC** |
| --- |

| **Line** | **Search term** | **Expanders/limiters** | **Results** |
| --- | --- | --- | --- |
| 1 | (DE "Electronic Learning" OR DE "Intelligent Tutoring Systems" OR DE "Interactive Video" OR DE "Computer Uses in Education" OR DE "Computer Assisted Instruction" OR DE "Computer Assisted Testing" OR DE "Computer Managed Instruction" OR DE "Integrated Learning Systems" OR DE "Online Courses" OR DE "Virtual Classrooms" OR DE "Virtual Schools" OR DE "Virtual Universities" OR DE "Web Based Instruction") | Expanders - Apply equivalent subjects  Search modes - Boolean/Phrase | 87138 |
| 2 | (Blackboard Learn or CERTPOINT or D2L or Desire2Learn or Brightspace or eCollege or Edmodo or EduNxt or Engrade or GlobalScholar or (Glow N20 Scott*) or HotChalk or Kahoot or Kannu or SAP or Skillsoft or Spongelab or SuccessFactors or "SumTotal Systems" or Taleo or (Teams N20 microsoft) or Uzity or aTutor or Chamilo or Claroline or Canvas or eFront or ILIAS or LAMS or "LON CAPA" or Moodle or "Open edX" or OLAT or OpenOLAT or Sakai or SWAD or WeBWorK or "e khool" or CallidusCloud or "Cornerstone OnDemand" or DoceboLMS or eFront or EthosCE or "Google Classroom*" or Grovo or "Growth Engineering" or "Halogen Software" or "Inquisiq R3" or "Learning Suite" or itslearning or Kannu or OpenLearning or Udutu or "ANGEL Learning" or Click2Learn or CourseInfo or "Learn com" or Elluminate or PeopleSoft or "Plateau Systems" or Softscape or SuccessFactors or WebCT or Instructure or Oracle or Docent or Zoom or coursera or futurelearn or iversity or "khan academy" or udemy or swayam or "mit ocw" or openclassroom*) | Expanders - Apply equivalent subjects  Search modes - Boolean/Phrase | 3916 |
| 3 | (elearn* or e-learn* or internet-based or web-based or virtual or online or computer-based or net-based or webinar* or video* or recorded or recording* or learning management system* or digital* or edutech* or education* tech* or synchronous or asynchronous or webcast* or screencast* or screenshar* or screen-shar* or mlearn* or m-learn* or web-conferenc* or computer simulat* or cd-rom* or vhs* or dvd*) | Expanders - Apply equivalent subjects  Search modes - Boolean/Phrase | 612166 |
| 4 | S1 OR S2 OR S3 | Expanders - Apply equivalent subjects  Search modes - Boolean/Phrase | 631688 |
| 5 | DE "Student Motivation" OR DE "Learning Motivation" | Expanders - Apply equivalent subjects  Search modes - Boolean/Phrase | 32075 |
| 6 | (motivat* N4 (student* or learner* or staff* or educat* or trainee* or instructee* or user? or pupil* or mentee* or tutee* or antendee* or participant*)) | Expanders - Apply equivalent subjects  Search modes - Boolean/Phrase | 39893 |
| 7 | TI motivat* OR SU motivat* | Expanders - Apply equivalent subjects  Search modes - Boolean/Phrase | 52809 |
| 8 | S5 OR S6 OR S7 | Expanders - Apply equivalent subjects  Search modes - Boolean/Phrase | 61655 |
| 9 | S4 AND S8 | Expanders - Apply equivalent subjects  Search modes - Boolean/Phrase | 24341 |
| 10 | (DE "Allied Health Occupations Education" OR DE "Clinical Teaching (Health Professions)" OR DE "Medical Education" OR DE "Graduate Medical Education" OR DE "Nursing Education" OR DE "Pharmaceutical Education" OR DE "Veterinary Medical Education" OR DE "Nursing Students") AND (DE "Dental Schools" OR DE "Medical Schools" OR DE "Medical Students" OR DE "Premedical Students") | Expanders - Apply equivalent subjects  Search modes - Boolean/Phrase | 4712 |
| 11 | (medic* or physician* or doctor* or pediatric* or paediatric* or allergist* or anesthesiologist* or dermatologist* or radiologist* or general practitioner* or geneticist* or neurologist* or obstetrician* or gynecologist* or gynaecologist* or ophthalmologist* or pathologist* or oncologist* or urologist* or surgeon* or nurs* or therapist* or dent* or pharmac* or allied health* or physiotherapist* or psychiatrist* or psychologist* or audiolog* or chirop* or podiatr* or chiropractor* or dietition* or dietetic* or hearing aid* or homeopath* or RMT or medic* laborator* or medical radiat* or radiographer* or midwif* or midwiv* or naturopath* or osteopath* or operating department* or optician* or optometr* or orthodont* or orthopt* or paramed* or prosthetic* or pedorthist* or orthotist* or respirator* or speech language or social work* or traditional chinese medic* or acupunctur*) | Expanders - Apply equivalent subjects  Search modes - Boolean/Phrase | 138703 |
| 12 | health* N4 (profession* or occupation* or personnel*) | Expanders - Apply equivalent subjects  Search modes - Boolean/Phrase | 20386 |
| 13 | hospital* N4 (staff* or profession* or personnel*) | Expanders - Apply equivalent subjects  Search modes - Boolean/Phrase | 925 |
| 14 | (health* or hospital*) N4 (trainee* or student*) | Expanders - Apply equivalent subjects  Search modes - Boolean/Phrase | 56791 |
| 15 | S10 OR S11 OR S12 OR S13 OR S14 | Expanders - Apply equivalent subjects  Search modes - Boolean/Phrase | 182078 |
| 16 | S9 AND S15 | Expanders - Apply equivalent subjects  Search modes - Boolean/Phrase | 1696 |
| 17 | S9 AND S15 | Limiters - Date Published: 19900101-20231231  Expanders - Apply equivalent subjects  Search modes - Boolean/Phrase | 1275 |

### **Web of Science (SSCI, AHCI, BHCI, ISTP, ESCI, SCI, BSCI, ISSHP)**

- WOS.SSCI: 1900 to 2022
- WOS.AHCI: 1975 to 2022
- WOS.BHCI: 2005 to 2022
- WOS.ISTP: 1990 to 2022
- WOS.ESCI: 2005 to 2022
- WOS.SCI: 1900 to 2022
- WOS.BSCI: 2005 to 2022
- WOS.ISSHP: 1990 to 2022

| Line | Search terms | Results |
| --- | --- | --- |
| 1 | (TS=((computer assisted OR elearn* or e-learn* or internet based or web based or virtual or online or computer based or net based or webinar or video* or recorded* or recording* or learning management system* or digital or blackboard learn* or certpoint or d2l or desire2learn or brightspace or ecollege or edmodo or edunxt or endgrade or globalscholar or hotchalk or kahoot or kannu or SAP or skillsoft or spongelab or successfactors or sumtotal systems or taleo or uzity or aTutor or chamilo or claroline or canvas or efront or ilias or lams or lon capa or moodle or open edx or olat or openolat or sakai or swad or webwork or e-khool or calliduscloud or cornerstone ondemand or docebolms or efront or ethosce or google classroom or microsoft teams or grovo or growth engineering or halogen software or inquisiq r3 or learning suite or itslearning or kannu or openlearning or udutu or angel learning or click2learn or courseinfo or learn com or elluminate or peoplesoft or plateau systems or softscape or successfactors or webct or instructure or oracle or docent or zoom or coursera or futurelearn or iversity or khan academy or udemy or swayam or mit ocw or openclassroom or edutech or synchronous or asynchronous or webcast* or screencast* or screenshar* or screen-shar* or mlearn* or m-learn* or web-conferenc* or computer simulation* or cd-rom or vhs or dvd))) | 4348985 |
| 2 | TS=(health personnel* or health occupation* or health professional* or hospital* personnel* or hospital* professional* or medic* or physician* or doctor* or pediatric* or paediatric* or allergist* or anesthesiologist* or anaesthesiologist* or dermatologist* or radiologist* or general practitioner* or geneticist* or neurologist* or obstetrician* or gyn?ecologist* or ophthalmologist* or pathologist* or oncologist* or urologist* or surgeon* or nurs* or therapist* or dent* or pharmac* or allied health* or physiotherapist* or psychiatrist* or psychologist* or audiolog* or chirop* or podiatr* or chiropractor* or dietition* or dietetic* or hearing aid* or homeopath* or RMT or medic* laborator* or medical radiat* or radiographer* or midwif* or midwiv* or naturopath* or osteopath* or operating department* or optician* or optometr* or orthodont* or orthopt* or paramed* or prosthetic* or pedorthist* or orthotist* or respirator* or speech language or social work* or traditional chinese medic* or acupunctur* or hospital staff* or clinic personnel* or clinic staff*) | 6490193 |
| 3 | TS=(motivat* NEAR (student* or teach* or educat* or school* or universit* or "professional development" or curricul* or competen* or pedagog* or learn* or instruct* or class or classes or workshop* or module* or train* or upskill* or retrain* or in-service* or inservice* or "continuing professional" or cpd or cme or "life-long learning" or "lifelong learning" or "certificate program" or "certificate programs" or certification* or trainee* or participant* or mentee* or tutee* or instructee* or attendee* or pupil*)) | 112007 |
| 4 | #3 AND #2 AND #1 | 5173 |
| 5 | #3 AND #2 AND #1 limited to 1990-01-01 – 2022-12-31 | 5172 |
