# Supplementary material for: Motivation Theories and Constructs in Experimental Studies of Online Instruction: Systematic Review and Directed Content Analysis
Source: JMIR Med Educ. 2025 Apr 11;11:e64179. doi: 10.2196/64179 (PMC12032500; doi:10.2196/64179)
Supplement: Multimedia Appendix 2 [file mededu_v11i1e64179_app2.docx]

## Searches run September 15, 2022 in:

- OFS Registries

| **Search** | **Search terms** | | | | **Results*** |
| --- | --- | --- | --- | --- | --- |
|  | **Web-based medium** | **Motivation** | **Learning** | **Learners** |  |
| 1 | “computer-assisted instruction” | motivat* | learn* | medic* | 8,796 |
| 2 | e-learn* OR elearn* | motivat* | learn* | medic* | 8,795 |
| 3 | virtual | motivat* | learn* | medic* | 9,393 |
| 4 | online | motivat* | learn* | medic* | 12,003 |
| 5 | “computer-assisted instruction” | motivat* | learn* | nurs* | 7.278 |
| 6 | e-learn* OR elearn* | motivat* | learn* | nurs* | 7,277 |
| 7 | virtual | motivat* | learn* | nurs* | 7,891 |
| 8 | online | motivat* | learn* | nurs* | 10,611 |
| 9 | “computer-assisted instruction” | motivat* | learn* | health | 13,224 |
| 10 | e-learn* OR elearn* | motivat* | learn* | health | 13,221 |
| 11 | virtual | motivat* | learn* | health | 13,773 |
| 12 | online | motivat* | learn* | health | 16,018 |

*Results filtered to only include OSF Registries
