# Supplementary material for: Motivation Theories and Constructs in Experimental Studies of Online Instruction: Systematic Review and Directed Content Analysis
Source: JMIR Med Educ. 2025 Apr 11;11:e64179. doi: 10.2196/64179 (PMC12032500; doi:10.2196/64179)
Supplement: Multimedia Appendix 3 [file mededu_v11i1e64179_app3.docx]

We extracted the following items: study title, first author, publication year, journal, geographic location in which the study was completed (if not stated, based on the authors’ institutional affiliations), health profession of participants, training status of participants, sample size, topic(s) of instruction, length of instruction, setting in which instruction was delivered to participants (e.g., remotely), whether instruction was embedded within participants’ regular experience (e.g., compared two versions of an existing course), device on which instruction was accessed (e.g., computer, mobile phone), technology used to deliver instruction (e.g., internet, CD-ROM), study design, use of motivational theory, use of motivational theory to guide certain aspects of the study, use of other (non-motivational) theory, motivational construct(s) targeted by instructional designs, definitions of motivational construct(s) (if applicable), other learning processes targeted by instructional designs (e.g., cognitive load), assessed outcome categories (e.g., motivation, SRL, achievement), description of outcomes, whether outcomes were naturally occurring through participants’ regular experience (e.g., outcome was an existing course exam), presence/details of mediation analyses, and presence/details of moderation analyses. The unit of analysis for this review was at the article level.
