# Supplementary material for: Motivation Theories and Constructs in Experimental Studies of Online Instruction: Systematic Review and Directed Content Analysis
Source: JMIR Med Educ. 2025 Apr 11;11:e64179. doi: 10.2196/64179 (PMC12032500; doi:10.2196/64179)
Supplement: Multimedia Appendix 4 [file mededu_v11i1e64179_app4.docx]

| **Construct category** | **Included constructs** | **Description** |
| --- | --- | --- |
| **Goals** | Achievement goals from AGT | Instructional designs targeting goals aim to orient learners toward different standards of competence (e.g., task-based, other-based) and/or for different purposes (e.g., to develop mastery, to demonstrate performance). |
| **Competence and control beliefs** | Expectancies for success from EVT, competence from SDT, self-efficacy and outcome expectancies from SCT, outcome attributions from Attribution Theory, control of learning beliefs from CVT, and confidence from the ARCS model | Instructional designs targeting competence and control beliefs aim to support learners’ beliefs that they can successfully learn the presented content, demonstrate a given performance, or achieve a given outcome, and/or that successful learning is under their control. |
| **Extrinsic value beliefs** | Attainment value and utility value from EVT, the taxonomy of extrinsic motivations from SDT, extrinsic value from CVT, and relevance from the ARCS model | Instructional designs targeting extrinsic value beliefs/motives aim to stimulate learners to engage with instruction to obtain outcomes external to instruction itself (e.g., future goals). |
| **Intrinsic value beliefs** | Enjoyment/interest value from EVT, intrinsic motivation from SDT, intrinsic value from CVT, and attention [essentially curiosity] from the ARCS model | Instructional designs targeting intrinsic value beliefs/motives aim to stimulate learners to engage with instruction out of feelings of interest, curiosity, and intrinsic satisfaction. |
| **Autonomy** | Autonomy from SDT | Instructional designs targeting autonomy aim to provide learners with more choice regarding whether and/or how to engage with instruction. |
| **Social connectedness** | Relatedness from SDT | Instructional designs targeting social connectedness aim to facilitate greater (and perhaps higher quality) interactions between learners and their peers and/or instructors. |
