# Supplementary material for: Motivation Theories and Constructs in Experimental Studies of Online Instruction: Systematic Review and Directed Content Analysis
Source: JMIR Med Educ. 2025 Apr 11;11:e64179. doi: 10.2196/64179 (PMC12032500; doi:10.2196/64179)
Supplement: Multimedia Appendix 5 [file mededu_v11i1e64179_app5.docx]

| **First author (year)** | **Summary of comparison(s)** | **Summary of theoretical underpinnings** | **Summary of design** |
| --- | --- | --- | --- |
| Allen (2008) | *Sample:* Medical students (*n* = 856)  *Topic:* Peripheral nervous system  *Design #1:* Course delivered via didactic lecture, dissection lab, web-based lecture notes, and web-based interactive learning objects (including patient cases, jeopardy games, simulated interactive patients, flashcards, and quizzes)  *Design #2:* Course delivered via didactic lecture, dissection lab, and PowerPoint lecture notes provided on a CD* | *Motivational theory:* None mentioned  *Motivational construct(s) targeted:* Intrinsic value beliefs/motives | Quasi-experimental trial |
| Berndt (2020) | *Sample:* Medical students (*n* = 100)  *Topic:* ECG interpretation  *Design #1:* Virtual patient cases with error analysis prompts (students asked to recognize, classify, and discuss how to prevent an error when one occurs) and justification prompts (students asked to justify their answers)  *Design #2:* Virtual patient cases with error analysis prompts  *Design #3:* Virtual patient cases with justification prompts  *Design #4:* Virtual patient cases with no prompts | *Motivational theory:* None mentioned  *Motivational construct(s) targeted:* None mentioned | Randomized parallel-groups trial |
| Blackmore (2006) | *Sample:* Psychotherapists and physiotherapists in practice (*n* = 167)  *Topic:* Unclear  *Design #1:* Collaborative online course, redesigned to promote greater use of the discussion forum  *Design #2:* Non-collaborative online course | *Motivational theory:* None mentioned  *Motivational construct(s) targeted:* Intrinsic value beliefs/motives | Quasi-experimental trial |
| Bock (2021) | *Sample:* Medical students (*n* = 51)  *Topic:* Aetiology and therapy of cleft lips and palates  *Design #1:* Instructional video with embedded ‘activating’ elements (drag-and-drop, matching, fill-in-the-blank, true/false, and short answer questions), consistent with the ‘sandwich principle’  *Design #2:* Instructional video without embedded elements | *Motivational theory:* None mentioned  *Motivational construct(s) targeted:* Autonomy | Randomized parallel-groups trial |
| Booth (2018)‡ | *Sample:* Nursing students (*n* = 120 is the recruitment target)  *Topic:* Electronic medical administration record systems  *Design #1:* Video and PowerPoint resources + a serious game (involved administering medications via an electronic system and receiving feedback on errors)  *Design #2:* Video and PowerPoint resources only | *Motivational theory:* None mentioned  *Motivational construct(s) targeted:* None mentioned | Randomized parallel-groups trial |
| Brull (2017) | *Sample:* Nurses in practice (*n* = 115)  *Topic:* Wound management, pain management, and fall prevention management  *Design #1:* Serious game that included avatars, points, quests, and challenges wrapped up in a fantasy narrative  *Design #2:* Online learning modules (PowerPoint presentations)* | *Motivational theory:* None mentioned  *Motivational construct(s) targeted:* None mentioned | Quasi-experimental trial |
| Buijs-Spanjers (2018) | *Sample:* Medical students (*n* = 156)  *Topic:* Caring for delirious patients  *Design #1:* Serious game (players experience 4 days as a caregiver, choosing different actions, and then the corresponding 4 nights as a patient)  *Design #2:* Two videos on delirium  *Design #3:* Video on healthy aging | *Motivational theory:* None mentioned  *Motivational construct(s) targeted:* None mentioned | Randomized parallel-groups trial |
| Buijs-Spanjers (2019) | *Sample:* Medical students (*n* = 157)  *Topic:* Caring for delirious patients  *Design #1:* Playing a serious game in ‘dark play mode’ (participants instructed to take actions to make delirium symptoms more serious)  *Design #2:* Playing a serious game in ‘normal play mode’ (participants instructed to provide good care) | *Motivational theory:* None mentioned  *Motivational construct(s) targeted:* None mentioned | Randomized parallel-groups trial |
| Cao (2018) | *Sample:* Dental students (*n* = 82)  *Topic:* Dental topics, specifics not reported  *Design #1:* Clinical simulation training course with a study announcement system (emails regarding students’ study levels and a summary of their learning records)  *Design #2:* Clinical simulation training course without a study announcement system | *Motivational theory:* None mentioned  *Motivational construct(s) targeted:* None mentioned | Quasi-experimental trial |
| Colonnello (2020) | *Sample:* Medical students (*n* = 130)  *Topic:* Minimally invasive mitral valve repair, robotic assisted lung resection  *Design #1:* Video with emotionally-relevant patient information  *Design #2:* Video with standard patient information | *Motivational theory:* Self-Determination Theory, Control-Value Theory  *Use of motivational theory:* Informed the research question; Informed the Experimental Conditions; Informed Any Methods or Measures; Informed the interpretation of results  *Motivational construct(s) targeted:* Social connectedness | Randomized cross-over trial |
| Cook (2009) | *Sample:* Physicians in postgraduate training (*n* = 124)  *Topic:* Asthma and depression  *Design #1:* Web-based module with an adaptive design (if a learner responded correctly to an introductory question, then they could skip detailed information)  *Design #2:* Web-based module with a non-adaptive design | *Motivational theory:* ARCS model  *Use of motivational theory:* Informed the research question; Informed the Experimental Conditions; Informed Any Methods or Measures; Informed the interpretation of results  *Motivational construct(s) targeted:* Intrinsic value beliefs/motives, Extrinsic value beliefs/motives | Randomized cross-over trial |
| Dankbaar (2016) | *Sample:* Medical students (*n* = 79)  *Topic:* Management of critically ill patients  *Design #1:* E-module (text with images, questions with feedback, and a video) plus high-fidelity cases packaged into a serious game with a narrative storyline and a leaderboard  *Design #2:* E-module plus low-fidelity (text-based) cases  *Design #3:* E-module only | *Motivational theory:* None mentioned  *Motivational construct(s) targeted:* Intrinsic value beliefs/motives | Randomized parallel-groups trial |
| Dankbaar (2017) | *Sample:* Medical students (*n* = 129)  *Topic:* Patient safety  *Design #1:* Serious game that included three parts: brief video lectures, biofeedback breathing exercises, and simulated patient care missions in a virtual flying hospital  *Design #2:* E-module that included written information that mirrored videos and written info on biofeedback* | *Motivational theory:* None mentioned  *Motivational construct(s) targeted:* None mentioned | Randomized parallel-groups trial |
| Dousay (2016) | *Sample:* Paramedical professionals in practice (*n* = 137)  *Topic:* Driver safety  *Design #1:* E-learning module with animations and spoken text  *Design #2:* E-learning module with animations and printed text  *Design #3:* E-learning module with animations, spoken text, and (summarized) printed text | *Motivational theory:* Self-Determination Theory, Four-Phase Model of Interest Development  *Use of motivational theory:* Informed the research question; Informed the Experimental Conditions; Informed Any Methods or Measures; Informed the interpretation of results  *Motivational construct(s) targeted:* Intrinsic value beliefs/motives | Randomized parallel-groups trial |
| Drees (2020) | *Sample:* Medical students (*n* = 213)  *Topic:* Medical histology (liver, gall bladder, pancreas)  *Design #1:* Interactive e-learning software with an auditory component and quiz questions  *Design #2:* Established software without auditory components or quiz questions* | *Motivational theory:* ARCS Model  *Use of motivational theory:* Informed the research question; Informed the Experimental Conditions; Informed Any Methods or Measures; Informed the interpretation of results  *Motivational construct(s) targeted:* Intrinsic value beliefs/motives, Extrinsic value beliefs/motives | Quasi-experimental trial |
| El Machtani El Idrissi (2022) | *Sample:* Nursing students (*n* = 58)  *Topic:* Pediatric nursing practices  *Design #1:* Content taught at a distance plus a serious game  Design #2: Content taught at a distance without a serious game | *Motivational theory:* ARCS Model  *Use of motivational theory:* Informed Any Methods or Measures  *Motivational construct(s) targeted:* Intrinsic value beliefs/motives, Extrinsic value beliefs/motives, Competence and control beliefs | Randomized parallel-groups trial |
| Frith (2003) | *Sample:* Nursing students (*n* = 174)  *Topic:* Cardiac rhythm interpretation  *Design #1:* Online course with mixed communication approach (included didactic materials and frequent online communication among students and between students and the instructor using chat rooms, email, and discussion forums)  *Design #2:* Online course with internal-only communication approach (didactic materials only) | *Motivational theory:* None mentioned  *Motivational construct(s) targeted:* Intrinsic value beliefs/motives | Randomized parallel-groups trial |
| Goldingay (2014) | *Sample:* Social work students (total *n* unclear; students who completed the evaluation survey = 32)  *Topic:* Social work practice skills  *Design #1:* Blended (but mostly online) course, students assigned to small groups and required to request peer-assessment, provide self-assessment for their mini role-play practice videos, and engage in online group cohesion exercises (e.g., sharing photos and answering questions about themselves)  *Design #2:* Blended (but mostly online) course, students assigned to small groups and invited to request peer-assessment and provide self-assessment for their mini role-play practice videos | *Motivational theory:* None mentioned  *Motivational construct(s) targeted:* Social connectedness | Quasi-experimental trial |
| Haftador (2021) | *Sample:* Nursing students (*n* = 89)  *Topic:* Basic nursing concepts  *Design #1:* Synchronous class  *Design #2:* Flipped jigsaw class (students sent videos beforehand and then engaged in synchronous jigsaw activities) | *Motivational theory:* Self-Determination Theory  *Use of motivational theory:* Informed the research question; Informed Any Methods or Measures; Informed the interpretation of results  *Motivational construct(s) targeted:* None mentioned | Randomized parallel-groups trial |
| Hedman (2013) | *Sample:* Medical students (*n* = 40)  *Topic: E*ndoscopy training  *Design #1:* Playing Half-Life PC game for 5 weeks in preparation for simulation training  *Design #2:* Playing Chessmaster PC game for 5 weeks in preparation for simulation training* | *Motivational theory:* Social Cognitive Theory, Engagement Modes Model  *Use of motivational theory:* Informed the research question; Informed Any Methods or Measures; Informed the interpretation of results  *Motivational construct(s) targeted:* Intrinsic value beliefs/motives, Competence and control beliefs | Randomized parallel-groups trial |
| Hwang (2020) | *Sample:* Nursing students (*n* = 56)  *Topic:* Intravenous injections using safety needles  *Design #1:* Game-based flipped learning (game included responding to normal and abnormal collecting scenarios)  *Design #2:* Video-based flipped learning | *Motivational theory:* None mentioned  *Motivational construct(s) targeted:* Intrinsic value beliefs/motives | Quasi-experimental trial |
| Inangil (2022) | *Sample:* Nursing students (*n* = 70)  *Topic:* Diabetes nursing  Design #1: Virtual lecture supplemented with animations (using Powtoon) and gamification (using Kahoot!)  *Design #2:* Typical virtual lecture (i.e., with a PowerPoint) | *Motivational theory:* None mentioned  *Motivational construct(s) targeted:* Intrinsic value beliefs/motives | Randomized parallel-groups trial |
| Jones (2021) | *Sample:* Pharmacy students (*n* = 54)  *Topic:* Introduction to drug information  *Design #1:* Video-based flipped classroom with quiz questions embedded in the videos  *Design #2:* Video-based flipped classroom with videos and separate quizzes (accessed via the LMS) | *Motivational theory:* None mentioned  *Motivational construct(s) targeted:* Intrinsic value beliefs/motives | Randomized parallel-groups trial |
| Karaksha (2013) | *Sample:* Pharmacy students (*n* = 79)  *Topic:* Mechanisms of action for drugs  *Design #1:* Supplementary e-tools (PowerPoints with narration and embedded questions) with reminder announcements for students to access the tools  *Design #2:* Supplementary e-tools (PowerPoints with narration and embedded questions) with no reminder announcements | *Motivational theory:* None mentioned  *Motivational construct(s) targeted:* None mentioned | Quasi-experimental trial |
| Koop (2021) | *Sample:* Medical students (*n* = 279)  *Topic:* Gross anatomy dissection of the anterior and posterior triangles of the neck  *Design #1:* Audiovisual manual (interactive, web-based video of a dissection with narration, printed text, and labelled structures, accessible via LMS)  Design #2: Manual with text and static illustrations (accessible via LMS)* | *Motivational theory:* None mentioned  *Motivational construct(s) targeted:* None mentioned | Cluster randomized trial |
| Lee (2015) | *Sample:* Nursing students (*n* = 86)  *Topic:* Health education methods  *Design #1:* Mobile phone-based discussion group to discuss the advantages and disadvantages of different methods  *Design #2:* Computer-based discussion group to discuss the advantages and disadvantages of different methods | *Motivational theory:* Information and Communication Technology Acceptance Model  *Use of motivational theory:* Was not judged as influencing any of the four coded research processes  *Motivational construct(s) targeted:* None mentioned | Randomized parallel-groups trial |
| Liu (2019, Study 1) | *Sample:* Medical students (*n* = 157)  *Topic:* Doctor-patient communication skills  *Design #1:* Videoconference-based communication skills training platform with autonomy supportive reminder email to review feedback  *Design #2:* Videoconference-based communication skills training platform with controlling reminder email to review feedback  *Design #3:* Videoconference-based communication skills training platform with neutral reminder email to review feedback | *Motivational theory:* Self-Determination Theory  *Use of motivational theory:* Informed the research question; Informed the Experimental Conditions; Informed Any Methods or Measures; Informed the interpretation of results  *Motivational construct(s) targeted:* Extrinsic value beliefs/motives | Randomized parallel-groups trial |
| Maag (2004) | *Sample:* Nursing students (*n* = 96)  *Topic:* Basic math review and medication dosage calculation  *Design #1:* Interactive multimedia modules (interlinked pages and interactive review questions)  *Design #2:* Multimedia modules (single webpage)* | *Motivational theory:* Social Cognitive Theory  *Use of motivational theory:* Informed the research question; Informed Any Methods or Measures  *Motivational construct(s) targeted:* Competence and control beliefs | Randomized parallel-groups trial |
| Mahnken (2011) | *Sample:* Medical students (*n* = 96)  *Topic:* Several radiology topics (chest, emergency, MRI, computerized tomography, pediatric, neuroradiology)  *Design #1:* Self-determined access to case-based e-learning modules covering several radiology topics  *Design #2:* Mandatory access to case-based e-learning modules covering several radiology topics (required to pass radiology internship)* | *Motivational theory:* Self-Determination Theory  *Use of motivational theory:* Informed the research question; Informed the Experimental Conditions; Informed the interpretation of results  *Motivational construct(s) targeted:* Intrinsic value beliefs/motives, Extrinsic value beliefs/motives | Cluster randomized trial |
| Metz (2022) | *Sample:* Dental students (*n* = 120)  *Topic:* Dental physiology  *Design #1:* Pre-recorded lectures with 8-12 embedded ‘active learning’ activities (interspersed every 10-15 minutes; e.g., match terminology, compare/contrast pathologies)  *Design #2:* Pre-recorded lectures with 3-4 embedded ‘active learning’ activities (interspersed every 30 minutes)  *Design #3:* Pre-recorded lectures with no embedded activities* | *Motivational theory:* None mentioned  *Motivational construct(s) targeted:* None mentioned | Quasi-experimental trial |
| Mohan (2017) | *Sample:* Physicians in practice (*n* = 368)  *Topic:* Trauma triage decisions  Design #1: Narrative-based video game (incorporated mystery, representative and non-representative cases, and puzzle mechanics)  Design #2: Standard training apps (mySTLS and Trauma Life Support MCQ Review) | *Motivational theory:* Theory of Narrative Engagement  *Use of motivational theory:* Informed the research question; Informed the Experimental Conditions; Informed the interpretation of results  *Motivational construct(s) targeted:* None mentioned | Randomized parallel-groups trial |
| Mohan (2018) | *Sample:* Physicians in practice (*n* = 320)  *Topic:* Trauma triage decisions  *Design #1:* Narrative-based video game  *Design #2:* Puzzle-based video game (oriented toward analogical reasoning)  *Design #3:* Standard training apps (based on mySTLS and Trauma Life Support MCQ Review apps)* | *Motivational theory:* Theory of Narrative Engagement  *Use of motivational theory:* Informed the research question; Informed the Experimental Conditions; Informed Any Methods or Measures; Informed the interpretation of results  *Motivational construct(s) targeted:* Intrinsic value beliefs/motives | Randomized parallel-groups trial |
| Pereira (2022) | *Sample:* Dental students (*n* = 33)  *Topic:* Prosthodontic dentistry or pediatric dentistry (depending on year of dental school)  *Design #1:* Quiz game (embedded in a blended format)  *Design #2:* Static PDF file (embedded in a blended format) | *Motivational theory:* None mentioned  *Motivational construct(s) targeted:* Intrinsic value beliefs/motives | Randomized parallel-groups trial |
| Peterson (2016) | *Sample:* Nursing students (*n* = 617)  *Topic:* Unclear (experiment implemented in a graduate nursing course)  *Design #1:* Asynchronous discussion forum with social interdependence (students were required to work together to craft a joint post), summarizing (students were required to post a short synthesis of main ideas), and scripts (rules for student interactions)  *Design #2:* Asynchronous discussion forum with social interdependence and summarizing  *Design #3:* Asynchronous discussion forum with social interdependence  *Design #4:* Synchronous discussion with social interdependence, summarizing, and scripts  *Design #5:* Synchronous discussion with social interdependence and summarizing  *Design #6:* Synchronous discussion with social interdependence  *Design #7:* Asynchronous discussion forum with summarizing and scripts  *Design #8:* Asynchronous discussion forum with summarizing  *Design #9:* Asynchronous discussion form with no summarizing | *Motivational theory:* Social Interdependence Theory  *Use of motivational theory:* Informed the research question; Informed the Experimental Conditions; Informed Any Methods or Measures; Informed the interpretation of results  *Motivational construct(s) targeted:* None mentioned | Cluster randomized trial |
| Pittenger (2010) | *Sample:* Healthcare professional students and undergraduate students (*n* = 218, undifferentiated)  *Topics:* Medical terminology, non-prescription pharmacology, prescription pharmacotherapy  *Design #1:* A required undergraduate online course on medical terminology that integrated a textbook, educational CD-ROM, and final exam  *Design #2:* A required graduate online course on medical terminology that integrated a textbook, educational CD-ROM, and final exam  *Design #3:* An optional undergraduate online course on non-prescription pharmacotherapy that integrated a textbook, online presentations, study guides, virtual pharmacy for self-assessment, and final exam  *Design #4:* An optional undergraduate online course on prescription pharmacotherapy that integrated online presentations, study guides, direct-to-consumer advertising reflection questions, and no final exam | *Motivational theory:* ARCS Model  *Use of motivational theory:* Informed the research question; Informed the Experimental Conditions; Informed Any Methods or Measures; Informed the interpretation of results  *Motivational construct(s) targeted:* Intrinsic value beliefs/motives, Extrinsic value beliefs/motives, Competence and control beliefs | Quasi-experimental trial |
| Rajan (2022) | *Sample:* Medical students (*n* = 32)  *Topic:* Stroke and cerebrovascular anatomy  *Design #1:* Case-based e-learning module with drag-and-drop, multiple-choice, and click-and-point activities  *Design #2:* Web page without interactivity or clinical cases, but otherwise containing identical information and images | *Motivational theory:* None mentioned  *Motivational construct(s) targeted:* Intrinsic value beliefs/motives | Randomized parallel-groups trial |
| Rondon-Melo (2016) | *Sample:* Speech-language and hearing science students (*n* = 36)  *Topic:* Anatomy and physiology of thr myofunctional orofacial system  *Design #1:* 2D serious game with a quiz format  *Design #2:* 3D computational models, integrating explanatory texts and animations* | *Motivational theory:* ARCS Model  *Use of motivational theory:* Informed the research question; Informed Any Methods or Measures; Informed the interpretation of results  *Motivational construct(s) targeted:* None mentioned | Randomized parallel-groups trial |
| Rudolphi-Solero (2021) | *Sample:* Medical students (*n* = 463)  *Topic:* Radiological anatomy and radiological signs of the thorax, abdomen, and musculoskeletal system  *Design #1:* Quiz-based competition in Second Life, voluntary participation, winners received various prizes  *Design #2:* Quiz-based competition in Second Life, mandatory participation, participants were successively eliminated and did not continue participating, winners received various prizes  *Design #3:* Quiz-based competition in Second Life, mandatory participation, participants were successively eliminated but were required to continue participating, winners received various prizes | *Motivational theory:* Self-Determination Theory  *Use of motivational theory:* Informed the research question; Informed the Experimental Conditions; Informed the interpretation of results  *Motivational construct(s) targeted:* Extrinsic value beliefs/motives | Quasi-experimental trial |
| Scales (2016) | *Sample:* Physicians in postgraduate training (*n* = 422)  *Topic:* Healthcare quality improvement  *Design #1:* Email-based quiz with a competitive element (participants were assigned to a team of fellow residents, given a rock band alias, and given feedback on relative standing via individual and team leaderboards)  *Design #2:* Email based quiz without a competitive element (participants were only given feedback on individual progress) | *Motivational theory:* None mentioned  *Motivational construct(s) targeted:* Social connectedness | Randomized parallel-groups trial |
| Seibert (2004) | *Sample:* Nurses in practice (*n* = 12)  *Topic:* Advanced health assessment  *Design #1:* Teleconferencing classroom designed to enhance real-world interaction, autonomy support, collaboration, and teacher interaction  *Design #2:* Teleconferencing classroom without enhancements | *Motivational theory:* Guthrie and Wigfield Engagement Model  *Use of motivational theory:* Informed the research question; Informed the Experimental Conditions; Informed the interpretation of results  *Motivational construct(s) targeted:* Intrinsic value beliefs/motives, Autonomy, Social connectedness | Randomized cross-over trial |
| Su (2017) | *Sample:* Nursing students (*n* = 102)  *Topic:* Clinical pathway of cardiac catheterization  Design #1: Mobile 3D game-based learning system  Design #2: Traditional e-learning module* | *Motivational theory:* ARCS Model  *Use of motivational theory:* Informed the research question; Informed the Experimental Conditions; Informed Any Methods or Measures  *Motivational construct(s) targeted:* Intrinsic value beliefs/motives, Extrinsic value beliefs/motives, Competence and control beliefs | Quasi-experimental trial |
| Sward (2008) | *Sample:* Medical students (*n* = 100)  *Topic:* Pediatrics  *Design #1:* A web-based, interactive modification of the Pediatric Board Game  *Design #2:* Computerized flash cards (a 400-question game database supplied identical content as the game) | *Motivational theory:* None mentioned  *Motivational construct(s) targeted:* Intrinsic value beliefs/motives | Randomized parallel-groups trial |
| Wang (2018) | *Sample:* Medical students (*n* = 49)  *Topic:* Nephrology  *Design #1:* Computer-based learning environment with a cognitive mapping approach (participants used a cognitive mapping tool to represent their problem-solving experience for each case)  *Design #2:* Computer-based learning environment with a note-taking approach (participants used a note-taking tool to report their problem-solving process) | *Motivational theory:* Self-Determination Theory, Control-Value Theory  *Use of motivational theory:* Informed the research question; Informed the Experimental Conditions; Informed Any Methods or Measures; Informed the interpretation of results  *Motivational construct(s) targeted:* Intrinsic value beliefs/motives | Randomized parallel-groups trial |
| Wingo (2015) | *Sample:* Physicians in postgraduate training (*n* = 127)  *Topic:* Hypertension, obesity, coronary heart disease, chronic obstructive pulmonary disease  *Design #1:* Asynchronous online module with motivationally-enhanced self-assessment questions (participants answered questions as if they were supervising a medical student in the clinic)  *Design #2:* Asynchronous online module with standard self-assessment questions | *Motivational theory:* Expectancy-Value Theory  *Use of motivational theory:* Informed the research question; Informed the Experimental Conditions; Informed Any Methods or Measures  *Motivational construct(s) targeted:* Intrinsic value beliefs/motives, Extrinsic value beliefs/motives | Randomized cross-over trial |
| Woelber (2012) | *Sample:* Dental students (*n* = 85)  *Topic:* Localized aggressive periodontitis, supplemented with general information about anamnesis, diagnosis, therapy, and maintenance  *Design #1:* E-learning module developed using easy-to-use software (allows a teacher to add narration to a PowerPoint)  *Design #2:* E-learning module developed using complex software (allows a teacher to add interactive elements such as drag and drops and interactive questions) | *Motivational theory:* None mentioned  *Motivational construct(s) targeted:* None mentioned | Randomized parallel-groups trial |
| Zwart (2022) | *Sample:* Nursing students (*n* = 44) and students from universities of applied sciences (*n* = 74)  *Topic:* Mathematical medication related to liquid medication, infusion of fluids, and solid medication  *Design #1:* Computer-based virtual learning environment (Second Life) with worked examples involving domain-specific math knowledge  *Design #2:* Computer-based virtual learning environment (Second Life) with worked examples involving regular thinking strategies  *Design #3:* Computer-based virtual learning environment (Second Life) with both types of worked examples  *Design #4:* Computer-based virtual learning environment (Second Life) with no worked examples | *Motivational theory:* Self-Determination Theory, Social Cognitive Theory  *Use of motivational theory:* Informed the research question; Informed Any Methods or Measures; Informed the interpretation of results  *Motivational construct(s) targeted:* Intrinsic value beliefs/motives, Competence and control beliefs | Randomized parallel-groups trial |

*Other conditions included in the study, but were not web-based

‡Study protocol (results not available)
