# Supplementary material for: Motivation Theories and Constructs in Experimental Studies of Online Instruction: Systematic Review and Directed Content Analysis
Source: JMIR Med Educ. 2025 Apr 11;11:e64179. doi: 10.2196/64179 (PMC12032500; doi:10.2196/64179)
Supplement: Multimedia Appendix 6 [file mededu_v11i1e64179_app6.docx]

| **First author (date)**  Risk of Bias Legend  (A) Random sequence generation  (B) Allocation concealment  (C) Similar baseline outcome measurements  (D) Similar baseline characteristics  (E) Incomplete outcome data  (F) Blinded outcome measurement  (G) Protection against contamination  (H) Selective reporting  ‡Study protocol | **Risk of Bias** | | | | | | | |
| --- | --- | --- | --- | --- | --- | --- | --- | --- |
|  | **A** | **B** | **C** | **D** | **E** | **F** | **G** | **H** |
| Allen (2008) |  |  |  |  |  |  |  |  |
| Berndt (2020) |  |  |  |  |  |  |  |  |
| Blackmore (2006) |  |  |  |  |  |  |  |  |
| Bock (2021) |  |  |  |  |  |  |  |  |
| Booth (2018)‡ |  |  |  |  |  |  |  |  |
| Brull (2017) |  |  |  |  |  |  |  |  |
| Buijs-Spanjers (2018) |  |  |  |  |  |  |  |  |
| Buijs-Spanjers (2019) |  |  |  |  |  |  |  |  |
| Cao (2018) |  |  |  |  |  |  |  |  |
| Colonnello (2020) |  |  |  |  |  |  |  |  |
| Cook (2009) |  |  |  |  |  |  |  |  |
| Dankbaar (2016) |  |  |  |  |  |  |  |  |
| Dankbaar (2017) |  |  |  |  |  |  |  |  |
| Dousay (2016) |  |  |  |  |  |  |  |  |
| Drees (2020) |  |  |  |  |  |  |  |  |
| El Machtani El Idrissi (2022) |  |  |  |  |  |  |  |  |
| Frith (2003) |  |  |  |  |  |  |  |  |
| Goldingay (2014) |  |  |  |  |  |  |  |  |
| Haftador (2021) |  |  |  |  |  |  |  |  |
| Hedman (2013) |  |  |  |  |  |  |  |  |
| Hwang (2020) |  |  |  |  |  |  |  |  |
| Inangil (2022) |  |  |  |  |  |  |  |  |
| Jones (2021) |  |  |  |  |  |  |  |  |
| Karaksha (2013) |  |  |  |  |  |  |  |  |
| Koop (2021) |  |  |  |  |  |  |  |  |
| Lee (2015) |  |  |  |  |  |  |  |  |
| Liu (2019, Study 1) |  |  |  |  |  |  |  |  |
| Maag (2004) |  |  |  |  |  |  |  |  |
| Mahnken (2011) |  |  |  |  |  |  |  |  |
| Metz (2011) |  |  |  |  |  |  |  |  |
| Mohan (2017) |  |  |  |  |  |  |  |  |
| Mohan (2018) |  |  |  |  |  |  |  |  |
| Pereira (2021) |  |  |  |  |  |  |  |  |
| Peterson (2016) |  |  |  |  |  |  |  |  |
| Pittenger (2010) |  |  |  |  |  |  |  |  |
| Rajan (2022) |  |  |  |  |  |  |  |  |
| Rondon-Melo (2016) |  |  |  |  |  |  |  |  |
| Rudolphi-Solero (2021) |  |  |  |  |  |  |  |  |
| Scales (2016) |  |  |  |  |  |  |  |  |
| Siebet (2004) |  |  |  |  |  |  |  |  |
| Su (2017) |  |  |  |  |  |  |  |  |
| Sward (2008) |  |  |  |  |  |  |  |  |
| Wang (2018) |  |  |  |  |  |  |  |  |
| Wingo (2015) |  |  |  |  |  |  |  |  |
| Woelber (2012) |  |  |  |  |  |  |  |  |
| Zwart (2022) |  |  |  |  |  |  |  |  |
